# 

**Published:** 1883-01

**Authors:** 


### CONTENTS.

| | Page. | | Pag |
|---|---|---|---|
| Ventilation | 1 | Some large Lenses | 17 |
| How to Sleep | 2 | When to Eat Meat | 17 |
| Simple Remedies | 3 | Facts worth Knowing | 18 |
| To the Public | 4 | Remedy for Catarrh | 18 |
| Change of Location for Health | 6 | The Railways of the World | 19 |
| Rabies | 6 | Objects and Methods of Medication | 19 |
| Vulgar Habits | 7 | A Coal Economizer | 20 |
| Diet | 8 | Migration of Fish | 21 |
| Digestibility of Oysters | 9 | Various Items | 21 |
| Cure for Ivy Poisoning | 9 | Healthy Homes | 22 |
| Fresh Meat from New Zealand | 10 | Mushroom and its Poison | 24 |
| Forced Feeding in Phthisis | 10 | Birth Rate in France | 25 |
| The Cure of Diabetes | 11 | Physical Exercise | 26 |
| Typhoid Fever | 12 | Solidified Tea | 28 |
| Remedy for Scrofulous Affections | 12 | Decay of Teeth | 28 |
| Hot and Cold Drinks | 13 | Feminine Deformity | 29 |
| Improvement in Chimneys | 14 | Dangerous Cosmetics | 30 |
| The Alaska Possibilities | 15 | A Russian Ice Palace | 31 |
| Bacteria | 16 | Meerschaum | 32 |

